# Integration of smart sensors and IOT in precision agriculture: trends, challenges and future prospectives

**DOI:** 10.3389/fpls.2025.1587869

**Published:** 2025-05-14

**Authors:** Sheikh Mansoor, Shahzad Iqbal, Simona M. Popescu, Song Lim Kim, Yong Suk Chung, Jeong-Ho Baek

**Affiliations:** ^1^ Phenomics Laboratory, Department of Plant Resources and Environment, Jeju National University, Jeju, Republic of Korea; ^2^ Department of Electronic Engineering, Faculty of Applied Energy System, Jeju National University, Jeju, Republic of Korea; ^3^ Department of Biology and Environmental Engineering, University of Craiova, Craiova, Romania; ^4^ National Institute of Agricultural Sciences, Rural Development Administration (RDA), Jeonju, Republic of Korea

**Keywords:** precision agriculture, smart sensors, IoT, big data, automation, challenges

## Abstract

Traditional farming methods, effective for generations, struggle to meet rising global food demands due to limitations in productivity, efficiency, and sustainability amid climate change and resource scarcity. Precision agriculture presents a viable solution by optimizing resource use, enhancing efficiency, and fostering sustainable practices through data-driven decision-making supported by advanced sensors and Internet of Things (IoT) technologies. This review examines various smart sensors used in precision agriculture, including soil sensors for moisture, pH, and plant stress sensors etc. These sensors deliver real-time data that enables informed decision-making, facilitating targeted interventions like optimized irrigation, fertilization, and pest management. Additionally, the review highlights the transformative role of IoT in precision agriculture. The integration of sensor networks with IoT platforms allows for remote monitoring, data analysis via artificial intelligence (AI) and machine learning (ML), and automated control systems, enabling predictive analytics to address challenges such as disease outbreaks and yield forecasting. However, while precision agriculture offers significant benefits, it faces challenges including high initial investment costs, complexities in data management, needs for technical expertise, data security and privacy concerns, and issues with connectivity in remote agricultural areas. Addressing these technological and economic challenges is essential for maximizing the potential of precision agriculture in enhancing global food security and sustainability. Therefore, in this review we explore the latest trends, challenges, and opportunities associated with IoT enabled smart sensors in precision agriculture.

## 1 Introduction

Traditional agricultural practices have been utilized for generations, leveraging local knowledge and techniques passed down through families. This small-scale farming often includes strategies such as crop rotation (Lundborg, 1990; Vasilescu et al., 2023). However, a significant drawback of traditional agriculture is its reliance on outdated methods, which can limit productivity and efficiency. Farmers may experience challenges such as lower crop yields stemming from rigid crop rotations and inadequate pest management techniques (Jain, 2012). Moreover, traditional practices are typically more vulnerable to climate variability, making crops susceptible to extreme weather events and pest outbreaks. As a result, these methods struggle to meet the growing demands of a global population, prompting a need for more sustainable and productive farming approaches (Janc et al., 2019). The shift to precision agriculture represents a viable solution, enhancing farming efficiency through technology and data analytics. This modern approach optimizes resource use, increases yields, and promotes sustainability (Charania and Li, 2020; Karunathilake et al., 2023) Precision agriculture enables real-time monitoring and targeted interventions, allowing farmers to better adapt to climate change and improve economic viability while minimizing environmental impacts compared to traditional practices (Lipper et al., 2014; Bogoviz et al., 2023; Gemtou et al., 2024).

Precision agriculture represents a significant advancement in the modern agricultural sector, highlighting the increasing need to enhance food production efficiency while simultaneously reducing environmental impacts (Işık et al., 2017; Evett et al., 2020; Wu et al., 2022). As global populations rise and the demand for food escalates, the agricultural landscape must adapt—this is where precision agriculture comes into play, utilizing advanced technologies to empower farmers and optimize operations (Afzaal et al., 2020; Wanyama et al., 2024). At its core, precision agriculture focuses on using data-driven approaches to inform agricultural practices. By harnessing technologies like the Internet of Things (IoT), artificial intelligence (AI), big data analytics, and cloud computing (Rodríguez et al., 2017; Abioye et al., 2020; Mansoor and Chung, 2024; Mansoor et al., 2024b; Sheikh et al., 2024), farmers can make informed decisions that lead to better resource utilization and improved crop yields. The benefits of precision agriculture extend beyond simple yield enhancements; they also encompass significant reductions in resource wastage, particularly water and fertilizers. This not only conserves water but also ensures that crops receive the precise amounts of water they need for optimal growth (Uztürk and Büyüközkan, 2024).

Agriculture 1.0 was defined using basic tools, manual and animal labor, and a heavy dependence on natural factors such as sunlight and rainfall. Farmers depended on their understanding of the land, weather patterns, and traditional farming techniques passed down through generations, typically from fathers to sons. This period was characterized by subsistence farming, where families grew just enough food to meet their own needs. The Industrial Revolution marked a significant transformation in agriculture, leading to what is known as the “Green Revolution” due to substantial increases in crop yield and productivity (Gagliardi et al., 2022; Liu et al., 2021; Zhai et al., 2020; Aggarwal and Verma, 2022). Agriculture 2.0 introduced machinery like tractors and harvesters, replacing manual labor and enhancing efficiency. This period also saw the rise of chemical fertilizers and pesticides, which boosted crop yields but negatively impacted the environment. Many innovations stemmed from the re-adaptation of mechanical and chemical industries that had previously catered to military needs during World War II. Additionally, breeding programs expanded through public universities, research institutes, and private companies (Aggarwal and Verma, 2022; Gagliardi et al., 2022).

Agriculture 3.0, often referred to as precision agriculture, utilized technology to enhance farming methods. It integrated GPS technology, remote sensing, and Geographic Information Systems (GIS) to gather data on soil conditions, crop health, and weather patterns. This information was used to create detailed maps, allowing for the targeted application of inputs like fertilizers, pesticides, and irrigation, which reduced waste and lessened environmental harm. During this period, public awareness grew regarding the environmental consequences of excessive fertilizer and chemical usage (Aggarwal and Verma, 2022; Gagliardi et al., 2022; Casavola and Gagliardi, 2012; Nargotra and Khurjekar, 2020).

The evolution of precision agriculture is often framed within the contexts of Agriculture 4.0 and Agriculture 5.0. Agriculture 4.0, described as the “Digital Revolution in Agriculture,” focuses heavily on the incorporation of sophisticated technologies to facilitate efficient agricultural practices (Maffezzoli et al., 2024). Agriculture 4.0 significantly enhances precision agriculture through a variety of technological advancements that improve efficiency, accuracy, and sustainability in farming practices. By utilizing IoT sensors, farmers can collect real-time data on conditions such as soil moisture, temperature, and crop health, allowing for informed decision-making (Zhai et al., 2020; Javaid et al., 2022). Additionally, big data analytics helps identify trends and make predictive assessments that optimize resource allocation. Drones equipped with multispectral cameras provide high-resolution aerial imagery, enabling remote monitoring of large fields and identifying areas that require attention. AI and machine learning (ML) further aid precision agriculture by analyzing data to predict outcomes and automate decision-making processes related to irrigation, fertilization, and pest control (Latino et al., 2022). Variable rate technology (VRT) allows for tailored applications of inputs like fertilizers and pesticides based on specific field characteristics, reducing waste and environmental impact. GPS technology enhances precision mapping and guides autonomous machinery, ensuring accurate operation in planting, harvesting, and resource application (Latino et al., 2022; Tenreiro et al., 2023). Moreover, Agriculture 4.0 fosters collaboration and connectivity among farmers through shared data, promoting integrated farming systems that improve overall management. By optimizing resource use and reducing carbon footprints, these advancements contribute to sustainable agricultural practices, demonstrating how Agriculture 4.0 is revolutionizing the efficiency and effectiveness of farming (Zambon et al., 2019; Zhai et al., 2020; Javaid et al., 2022).

Transitioning into Agriculture 5.0, we observe a paradigm shift towards a more human-centric approach in agricultural innovation. While Agriculture 4.0 emphasizes data and automation, Agriculture 5.0 combines technology with human ingenuity and sustainable practices (Islam et al., 2024). It accommodates the use of advanced IoTs, robotics, AI, and collaborative efforts between humans and machines, promoting resilience within agricultural systems (Hurst and Spiegal, 2023; Kazakis and Tsirliganis, 2023). Agriculture 5.0 seeks to foster a deeper collaboration among human expertise, machine efficiency, and sustainable methodologies, creating a synergistic effect where both humans and machines can contribute to overcoming agricultural challenges (da Silveira and Amaral, 2022; Ross and Maynard, 2021). For example, autonomous agricultural equipment equipped with AI can work alongside farmers, enhancing their capabilities in tasks such as planting, harvesting, and pest control, while ensuring that these processes are executed with minimal environmental impact. Moreover, this evolution also accentuates the importance of sustainable practices in agriculture (Pham et al., 2013; de la Parte et al., 2024; Popescu et al., 2024). As awareness of environmental concerns grows, there is an increasing emphasis on practices that not only increase yield but also maintain ecological balance. By incorporating sustainable techniques within the precision agriculture framework, farmers can reduce their carbon footprint, enhance biodiversity, and maintain healthier soils (Fraser and Campbell, 2019; Juwono et al., 2023; Ku et al., 2023; Mansoor et al., 2024a). Precision agriculture is transforming agriculture by integrating advanced technologies to meet food production demands. This shift from a data-driven model to a collaborative approach, incorporating multi-omics data analysis, emphasizes the synergy between technology and human expertise, fostering resilient agricultural systems (Abioye et al., 2023). As stakeholders adopt these innovations, including the integration of multi-omics insights, the agricultural sector is increasingly poised to ensure food security and sustainability for the growing global population.

As environmental concerns grow, there is an increasing emphasis on agricultural practices that enhance yield while maintaining ecological balance. By integrating sustainable techniques within the precision agriculture framework, farmers can effectively reduce their carbon footprint, bolster biodiversity, and promote healthier soils (Konfo et al., 2024). Precision agriculture optimizes resource use and minimizes waste, leading to significant environmental benefits. For instance, reductions in carbon emissions result from decreased fertilizer and pesticide application, which not only lowers greenhouse gas emissions but also enhances soil health, measurable through a soil health index that reflects improved organic matter and nutrient availability (Ahmad and Dar, 2020; Farooqui et al., 2024). Moreover, precision irrigation enhances water use efficiency, conserving vital resources and protecting local ecosystems. Collectively, these metrics illustrate how precision agriculture fosters sustainable farming methods and enhances overall environmental stewardship.

IoT is a global network that enables devices to operate, identify, and monitor objects across the globe via the internet, connecting virtual and physical entities through integrated information and communication technologies (Pham et al., 2013; Abioye et al., 2023) The main objective of smart farming is to enhance real-time information sharing across autonomous networks using smart sensors and internet connectivity. Various communication solutions, such as wireless sensors and Radio-Frequency Identification (RFID) technologies, support interconnectivity among networks and devices (Juwono et al., 2023; Ku et al., 2023). In smart farming, critical parameters are monitored to improve yield, optimize environmental conditions, manage irrigation, control pests and fertilizers, oversee soil health, and enhance greenhouse production, all while reducing operational costs (Evans, 2011; Gómez Romero et al., 2016; Nukala et al., 2016; Fraser and Campbell, 2019; Mansoor et al., 2024a). These technologies play a vital role within IoT platforms and are classified into data acquisition, investigation, and evaluation categories (Zecha et al., 2013; Freeman and Freeland, 2015; Balafoutis et al., 2017). Countries such as those in Europe, Australia, and the USA have embraced smart farming, alongside individual nations like Italy (Borgogno Mondino and Gajetti, 2017), Brazil (Pivoto et al., 2018), Ireland (Das et al., 2019), and India (Mogili and Deepak, 2018).

**Figure 1:**
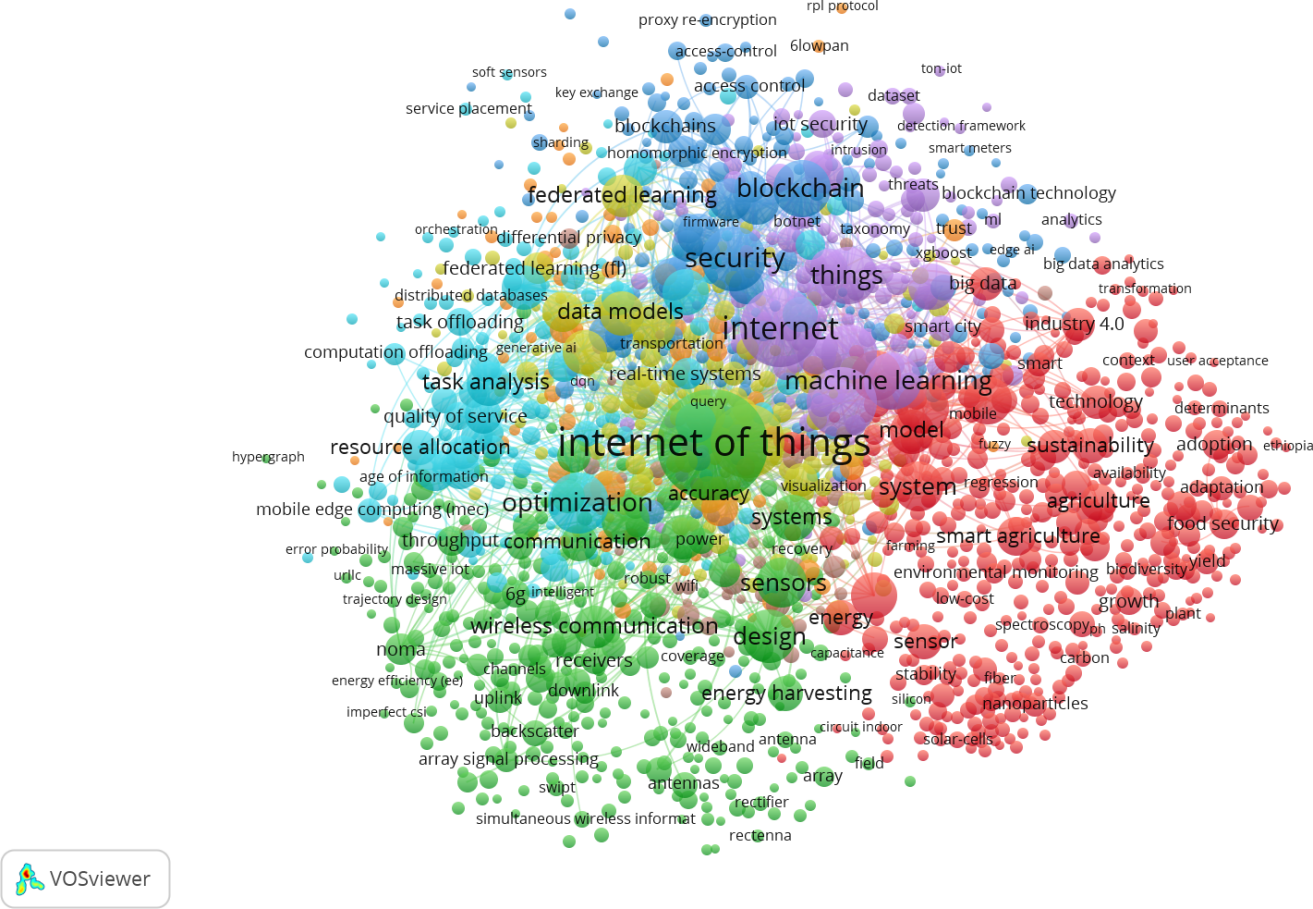
VOS viewer keyword co-occurrence network visualizes associations and clusters related to the “Internet of Things” (IoT). It features various color-coded clusters representing interconnected concepts. The central term, “Internet of Things,” connects to multiple branches, highlighting its relevance. Key clusters include the green cluster with “optimization” and “analytics,” focusing on data processing; the blue cluster with “security” and “blockchain,” emphasizing secure data transactions; the yellow cluster with “machine learning,” showcasing AI advancements; and the red cluster with “energy” and “sustainability,” addressing environmental concerns. Lines between keywords illustrate co-occurrence, helping identify trending topics and common research areas in IoT. This image was created with VOSviewer.

We analyzed the existing literature on the application of IoT platforms and wireless communication technologies across various agricultural activities. At the outset of this review, relevant articles were sourced from the Web of Science using keywords such as *“smart agriculture,” Agriculture 4.0, IoT, smart farming, digital agriculture.* This collection included 6000 recent articles comprehensive bibliometric information, which was subsequently used as input for VOS viewer (Software) analysis. This analysis assesses the frequency of keyword usage and citation metrics in the selected articles. Additionally, it visualizes the co-occurrence of keywords and the co-citation of references (**Figure 1**).

**Figure 2:**
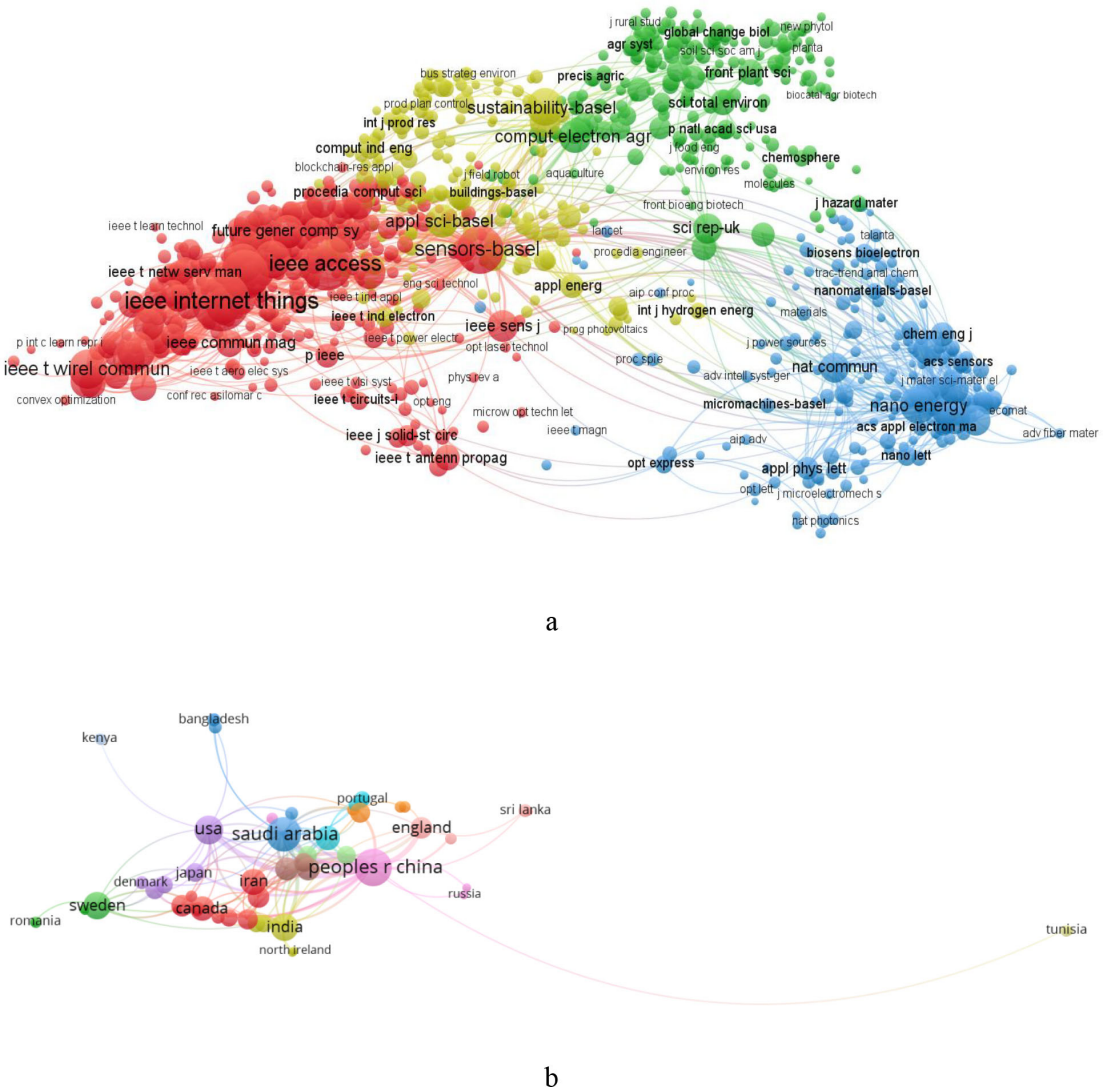
Network visualization of keyword usage and co-citation metrics in the journals. **(a)** Network visualization of co-citation of references across different journals, highlighting the interconnectedness of cited works. **(b)** Network visualization map illustrating international research in precision agriculture, showcasing the global distribution and collaboration in this field.

To create the visual map, specific criteria were established, with a minimum threshold of five co-occurrences for keywords. Out of a total of 20767 keywords, only 1440 met this threshold criterion. The keyword with greatest total link strength is selected. The size of each node represents the frequency of co-occurrences of terms, while the thickness of the connecting lines indicates how often these keywords appear together (**Figure 1**). The connections illustrate the relationships between items, and each node reflects the strength of a particular item. Similarly, co-citation analysis evaluates how frequently an article has been cited across the selected documents. The visualization of the co-occurrence network of citations is depicted in **Figure 2**.

## 2 Smart sensor trends

The modern agricultural industry has significant challenges that need the use of innovative technologies. Agriculture, a vital economic sector (Gollin, 2010) is being impacted by inflation, rising labor costs (Ray et al., 2023), and climate change (Outhwaite et al., 2022; Verma et al., 2022), resulting in diminished agricultural yields. In this scenario, Precision Agriculture emerges as a crucial solution. This sophisticated agricultural technique employs state-of-the-art technology to enhance the amount and timing of inputs necessary for cultivation, aiming to increase output and efficiency.

Sensors play a crucial role in agriculture, detecting environmental changes and transmitting information to processors (Ullo and Sinha, 2021; Liu et al., 2022). In precision agriculture, smart sensors integrate onboard computing capabilities, allowing them to process and analyze data independently (Rahman et al., 2023; Rajak et al., 2023; Saqib et al., 2024). These sensors are equipped with microprocessors that enable local data processing, autonomous decision-making, and direct communication with other devices via Wi-Fi, Bluetooth, or cellular networks (Yin et al., 2021). This autonomy is particularly useful in large-scale farming, where real-time data on soil conditions, plant health, and climate can significantly influence management decisions. Precision agriculture utilizes various sensor technologies to improve the efficiency and productivity of agricultural practices. Soil sensors deliver critical data on moisture levels, pH, temperature, nutrient content, and electrical conductivity, guiding decisions related to irrigation, fertilization, and planting (Roper et al., 2021). Plant health monitoring employs sensors like leaf sensors, chlorophyll fluorescence sensors, Normalized difference vegetation index (NDVI) sensors, and hyperspectral sensors (Li et al., 2021), while environmental sensors monitor atmospheric conditions that impact agricultural decisions (Rahman et al., 2024; Yin et al., 2021; Han, 2024). Specialized sensors, like yield monitors and water potential sensors, offer precise data on crop yield and soil water availability, enhancing farming operations (**Table 1**).

**Table 1:** Sensors used in precision agriculture, detailing their types, functions, and applications.

Sensor/Device	Type	Function	Application
DHT11	Temperature/Humidity Sensor	Monitoring Temperature & Humidity	Crop monitoring and environmental control
Capacitive Soil Moisture Sensor	Soil Sensor	Measuring Soil Moisture	Irrigation management and soil health
BMP180	Pressure Sensor	Weather Monitoring	Weather prediction and climate analysis
LM35	Temperature Sensor	Measuring Temperature	Crop health monitoring
SI1145	Light Sensor	Measuring UV Light & IR	Plant growth monitoring
GPS Module	GPS Sensor	Location Tracking	Precision farming
MQ-135	Air Quality Sensor	Air Quality Monitoring	Pest detection and environmental monitoring
Raspberry Pi	IoT Platform	Data Processing & Communication	Data aggregation for multiple sensors
Arduino	IoT Platform	Data Acquisition	Custom sensor integration
LoRaWAN Module	Communication Device	Long-range Data Transmission	Remote data transmission for farms

The cost of sensors is a crucial factor because it directly affects farmers’ ability to invest in these technologies within their budget constraints. The cost-effectiveness of agriculture sensors depends on their complexity and function (Saqib et al., 2020). Soil moisture sensors range from $50 to $300, while weather stations can cost $100 to several thousand dollars. Nutrient sensors typically range from $500 to $2000. Pest and disease sensors, part of advanced systems, also cost a few hundred to several thousand dollars. Agricultural drones vary from $1,000 to $25,000 or more, depending on features. Basic moisture sensors are inexpensive and provide essential data for irrigation management, while advanced sensors like electrical conductivity and nutrient sensors require higher initial investments but enable precise fertilization and soil health monitoring (Panagopoulos et al., 2014; Kiropoulos et al., 2021; Guerrero et al., 2021). Specialized sensors, like yield monitors and water potential sensors, help farmers assess crop performance and optimize water use, leading to improved crop quality and higher revenues. The return on investment of precision agriculture sensors depends on the scale of the farming operation, the type of sensor, and the specific needs of the farm. Low-cost sensors offer immediate benefits, while high-end technologies provide long-term savings through resource efficiency and yield optimization. Farmers should make informed decisions about which sensors align with their financial capabilities and operational goals.

### 2.1 Soil sensors

A soil sensor is a device used to measure various physical and chemical properties of soil, such as moisture content, temperature, pH level, electrical conductivity, and nutrient concentration (Yin et al., 2021). Traditional assessment methods, such as soil sampling and laboratory analyses, often lack the necessary spatial and temporal resolution. Therefore, there is an increasing demand for innovative technologies capable of providing precise soil data to enhance smart or precision agriculture systems (Zhang et al., 2024). Recent developments in soil sensors for precision agriculture focus on essential factors for monitoring plant growth cycles. Key elements affecting crop productivity include soil moisture, temperature, pH, nutrient levels, pests, and pollutants. Site-specific management practices, like irrigation and fertilizer usage, rely on data collected from various soil sensors (Kalita et al., 2017; de Jong et al., 2020). A review of six types of soil sensors highlights their technologies, designs, performance, advantages, and disadvantages as well as also discusses research trends and challenges in soil sensors and smart agriculture to guide future studies.

#### 2.2.1 Soil moisture sensors

Soil moisture is vital for assessing soil health and is key to plant growth. It affects the soil’s physical and chemical properties, which in turn impacts salt dissolution, the uptake of water and nutrients by plants, and the activity of microorganisms in the soil (Zhang et al., 2022). Keeping track of soil moisture levels is essential to ensure the right conditions for agricultural production. Soil moisture sensors help farmers measure the water content in the soil, allowing them to determine the optimal timing and amount of irrigation needed for healthy plant growth. Soil moisture measurement is crucial for various applications, including agriculture and hydrological studies. Techniques have been developed to measure soil moisture based on accuracy, cost, and complexity (Zhang et al., 2017, 2024).

Soil moisture sensors are categorized based on the technology they use to detect the moisture levels in the soil, and each type serves specific purposes. Volumetric sensors, particularly capacitive soil dielectric permittivity sensors, are common and appropriate for low-cost, wireless applications. Capacitive sensors measure the capacitance between two plates, which changes with the soil’s dielectric constant, influenced by its moisture content. These sensors are preferred for their low power requirements and minimal interference from soil salinity. Anindita Kalita’s study on polymethyl methacrylate (PMMA) coated capacitive sensors for soil moisture sensing suggests they could be a cost-effective and easy-to-fabricate solution for real-time soil moisture monitoring in agriculture. Despite initial sensitivity limitations, they could optimize irrigation practices and crop productivity, highlighting the need for further research (de la Parte et al., 2024).

Resistive sensors measure the electrical resistance between electrodes inserted into the soil, but their accuracy can be compromised by soil composition variations. Steven M. de Jong’s study on Electrical Resistivity Tomography (ERT) showed it can effectively monitor soil moisture dynamics under controlled field conditions, but challenges remain due to environmental variables and ERT’s limitations (de Jong et al., 2020).

Time Domain Reflectometry (TDR) and Time Domain Transmissometer (TDT) sensors use electromagnetic waves to measure moisture, however they are costly and complicated, suitable for research and precision agricultural activities. Time-Domain Reflectometry (TDR) is known for its precision and is widely used in scientific research. Zhongdian and his research group developed a TDR-based method for measuring soil erosion and soil moisture content, demonstrating high accuracy and automation potential. Further refinement and testing are suggested for wider applicability (Zhang et al., 2022) Time Domain Transmissometer (TDT) measures the transmission time of electromagnetic waves through the soil, providing excellent accuracy and usefulness for depth-specific moisture profiling. Raphaël Pederiva and colleagues developed an on-chip terahertz (THz) characterization technique for low-volume or thin-film materials. The method uses time-domain transmissometer to determine the complex refractive index of materials over a frequency range of hundreds of gigahertz (GHz). The device uses ultrafast photoconductive switches driven by a femtosecond laser, allowing for high precision and minimal sample volume (Krzeminska et al., 2022).

Frequency Domain Reflectometry (FDR) uses the frequency change of an electromagnetic wave to determine soil moisture, making it effective for continuous monitoring across various soil conditions. The study in the Gryteland catchment in Norway used frequency domain reflectometry (FDR) and electrical resistivity tomography (ERT) to monitor soil moisture and temperature patterns. Key findings showed different patterns on north-facing and south-facing slopes, impacting freezing and thawing cycles. The study suggests that local terrain features, particularly slope aspects, are crucial in hydrological processes and should be considered in environmental and agricultural management strategies (Ma et al., 2022).

Optical methods, such as visible and near-infrared spectrophotometry, leverage the soil’s light absorption and scattering properties, which change with moisture content. These methods are advantageous for non-contact measurements and are particularly useful in remote sensing applications (Abdulraheem et al., 2023). The choice of a suitable technique depends on the specific requirements of the application, including accuracy, cost considerations, and environmental conditions. The use of Sentinel-2 imagery and the optical trapezoid model (OPTRAM) to monitor soil moisture variability in agricultural production stages. The method, which uses Sentinel-2 imagery, is used to explore high-resolution spatial heterogeneity of soil moisture and monitor various stages of agricultural production (Hassanpour et al., 2020; Stańczyk et al., 2023). The results show that the OPTRAM model can produce accurate soil moisture estimates, improving irrigation management and crop growth understanding, ultimately leading to better water resource management in agriculture (Crioni et al., 2025).

Precision irrigation uses soil moisture sensors to monitor real-time water levels and optimize crop decisions. Sensors are deployed at multiple depths to capture moisture variations across the root zone (Bwambale et al., 2022). IoT-based systems analyze this data, combining weather forecasts and crop models to determine precise irrigation schedules. Automated systems adjust water application based on the data, ensuring efficient water distribution and preventing over- or under-irrigation (Kanimozhi and Vadivel, 2024). This dynamic approach refines irrigation strategies, conserving water, enhancing plant health, and improving agricultural yields. For example, in Zhang et al. (2017), an IoT-based soil monitoring system was implemented in a citrus orchard, where real-time soil moisture data helped optimize fertilization and irrigation strategies, leading to reduced water waste, improved efficiency, and enhanced crop productivity (Zhang et al., 2017).

#### 2.2.2 pH sensors

Soil pH is crucial for plant growth and fertilizer application efficiency. Real-time soil pH sensors, integrated with precision agriculture technologies, provide real-time feedback on soil conditions. These sensors communicate with cloud-based systems, enabling automated pH adjustments (**Table 1**) (Lavanaya and Parameswari, 2018; Yin et al., 2021; Fauziah et al., 2024).

In addition to improving nutrient absorption, real-time pH monitoring enhances soil microbial activity, which is vital for organic matter decomposition and natural nitrogen fixation (Xu et al., 2024). Studies have shown that microbial communities thrive best within a neutral to slightly acidic pH range (5.5–7.5), where beneficial bacteria such as nitrogen-fixing Rhizobia and phosphate-solubilizing Pseudomonas species actively contribute to soil fertility (Saeed et al., 2021). Savich investigated the application of soil amendments combined with pH-responsive sensors in saline soils, demonstrating that integrating real-time monitoring with phosphogypsum and organic fertilizers significantly enhanced CO_2_ assimilation and crop biomass (Savich et al., 2021). The study emphasized the role of real-time pH adjustments in improving photosynthetic activity, ultimately leading to higher productivity in challenging soil conditions.

Furthermore, Zhao analyzed the long-term impact of no-till (NT) agriculture on soil pH stability, revealing that real-time pH monitoring could help mitigate soil acidification by optimizing nitrogen application rates and periodic liming schedules. By integrating pH sensors into conservation tillage practices, farmers can maintain soil health while minimizing the negative effects of prolonged nitrogen fertilization (Zhao et al., 2022).

In addition to chemical amendments, biological strategies for pH optimization are gaining traction. Yaghoubi Khanghahi highlighted the potential of plant growth-promoting bacteria (PGPB) in modifying soil pH and improving nutrient availability. Their research demonstrated that combining bio-inoculants with real-time pH sensors allowed farmers to adjust pH levels in response to microbial activity, reducing dependence on synthetic fertilizers. The findings suggest that a holistic approach integrating biological, chemical, and technological solutions can maximize soil fertility and improve overall crop resilience (Yaghoubi Khanghahi et al., 2021). A typical conductometric pH sensor consists of conductivity electrodes and a thin layer of pH-responsive sensing material.

The study presents a 3D macroporous graphene-functionalized soil pH microsensor, fabricated on Si/SiO2 substrates with Au-interdigitated electrodes. The sensor increases conductance with pH increase, exhibiting a sensitivity of 97 μS/pH and a response of 650%. It detects soil pH variations in different soil samples, with sensitivity varying with gravimetric moisture contents (Penn and Camberato, 2019; Siddiqui and Aslam, 2023). A potentiometric soil pH sensor measures soil pH by detecting voltage difference between reference and pH-sensitive electrodes. Accurate pH readings are crucial for agriculture and research, requiring regular calibration and calibration with known solutions (Siddiqui and Aslam, 2023). Matthew McCole, presents a potentiometric measurement system for on-site soil pH and potassium levels detection (McCole et al., 2023). The system uses 3D printed ion-selective electrodes, a PSoC4 microcontroller, and a reference electrode for ion activity. The system is portable, user-friendly, and efficient, enabling real-time soil analysis and precise management of soil nutrients, potentially leading to better crop yields and reduced environmental impact (Childs et al., 2000). Ion-selective pH sensors (ISE) measure hydrogen ions in solutions or soil, providing accurate real-time data through a glass electrode and reference electrode, requiring regular calibration. An alternative method is the ion-selective field-effect transistor (ISFET), which includes a drain, source, and gate electrode. pH-sensitive materials like silicon oxide, silicon nitride, and aluminum oxide are coated on the gate electrode. When in contact with the solution, these materials induce changes in gate voltage, affecting the current between the source and drain electrodes based on pH variations. Due to the complexity of soil, ISFETs must be well-protected to avoid damage during insertion (Shylendra et al., 2025).

#### 2.2.3 Temperature sensors

Soil temperature, which varies between -10 and 50°C, is a significant determinant in agriculture, affecting germination, flowering, decomposition, and multiple phases of plant development (Bollero et al., 1996; Onwuka and Mang, 2018). It profoundly affects the physical, chemical, and microbiological processes in soil that are critical for plant growth. Soil temperature is influenced by factors such as specific heat capacity, thermal conductivity, bulk density, texture, water content, and surface coverings (Passioura, 2002; Hatfield and Prueger, 2015).

A soil temperature sensor operates by converting temperature fluctuations into an electrical signal, which is then processed into digital data. Various types of electronic temperature sensors suitable for this application include thermocouples, resistance temperature detectors (RTDs), thermistors, and semiconductor-based sensors (Davaji et al., 2017). Thermocouples function by generating a voltage due to the temperature difference at the junction of two dissimilar metals, typically iron and constantan. They are recognized for their rapid response and automation capabilities, making them appropriate for monitoring soil temperature. Specially calibrated cables are essential for long-distance measurements. Resistance temperature detectors (RTDs) are composed of a conductive metal wire coiled around a non-conductive core, offering high accuracy and stability. They display increased delicacy relative to thermocouples and show a reduced response time to temperature changes due to their protective housing (Kool et al., 2016).

Thermistors, made from ceramic or polymer materials, demonstrate a change in resistance when subjected to temperature fluctuations. They provide high resolution due to significant thermal coefficients; however, they require complex calibration because of their non-linear response. The resolution of this issue can be achieved (Xu et al., 2023). Kool and colleagues’ study highlights the importance of accurately measuring soil temperature gradients to determine soil heat and latent heat fluxes. They used thermistors to monitor soil temperature, but found discrepancies of 0.2°C under uniform conditions. To improve accuracy, they developed an *in-situ* calibration technique that minimized uncertainty to 0.05°C. This allowed for more precise measurements in a vineyard under arid conditions and showed stable thermistor offsets over a five-year period (Kool et al., 2016).

#### 2.2.4 Nutrient sensors

Nutrient sensors for soil are advanced tools critical for precision agriculture, designed to identify and measure essential soil nutrients, including nitrogen, phosphorus, and potassium. These sensors operate on various principles, including ion-selective electrodes, optical sensors, electrochemical sensors, and spectroscopy, each tailored for specific nutrient types (Burton et al., 2020a). They enable real-time monitoring and mapping of soil nutrient levels, thus facilitating precise and efficient fertilizer application. This approach improves crop yields and optimizes fertilizer use, reducing costs and minimizing environmental impacts by preventing nutrient runoff, thus preserving soil health and protecting water quality. Nutrient sensors represent a significant advancement in agricultural technology, enhancing sustainable farming practices by optimizing plant growth and resource management (Horváth et al., 2024).

Various nutrient sensors have been developed for agricultural applications, each demonstrating unique capabilities and stages of development (Burton et al., 2020b). The Visible-Near Infrared (Vis-NIR) sensor is currently in use in both laboratory and field settings, effectively measuring soil pH and nutrient levels. Similarly, the Visible-Mid Infrared (Vis-MIR) sensor has shown promise in laboratory settings for assessing soil mineral nitrogen content (Ehsani et al., 1999, 2001). The Attenuated Total Reflectance (ATR) spectroscopy sensor operates in both laboratory and field environments, focusing on soil nutrient analysis (Christy et al., 2003).

Raman spectroscopy, used in laboratory and field applications, is effective for evaluating various soil nutrients (Jahn et al., 2006). Additionally, Ion Selective Electrodes (ISE) and Ion-Selective Field Effect Transistors (ISFET) are utilized in laboratory and field settings for measuring soil pH and nutrients (Sudduth et al., 1997; Adamchuk et al., 2005; Aravamudhan and Bhansali, 2008; van Staden et al., 2018). Each sensor type contributes vital insights into soil health and nutrient management, enhancing precision agriculture practices (Burton et al., 2020b).

#### 2.2.5 Electrical conductivity sensors

Soil electrical conductivity (EC) is a crucial indicator in agriculture, as high soil salinity can negatively impact crop growth and reduce agricultural productivity. Soil EC is directly related to the types and concentrations of ions in soil moisture, such as sodium, chloride, calcium, and magnesium, which enhance the soil’s electrical conductivity. Higher moisture levels generally increase soil EC because they facilitate the movement of soluble salts (Liu et al., 2024). The physical composition of the soil, including its clay, sand, and organic matter content, can also influence EC readings. Clay soils typically have higher EC values due to their finer texture and greater cation exchange capacity.

Techniques for measuring soil EC include laboratory measurements, *in-situ* sensors, and remote sensing techniques. High levels of soil salinity can lead to osmotic stress, which can cause dehydration and stunted growth, and ion toxicity, which can accumulate in plant tissues to toxic levels. To mitigate these effects, various soil amendments and management practices may be employed, such as leaching, which involves applying ample irrigation water to flush out excess salts from the root zone, soil amendments, and crop selection and rotation (Wang et al., 2025).

#### 2.2.6 Soil pollutant sensors

Soil pollutant sensors are essential tools in modern agriculture and environmental management, designed to detect and measure harmful substances in the soil, such as heavy metals, pesticides, herbicides, and industrial pollutants. Excessive application of agrochemicals, industrial activities, and household waste contribute significantly to soil health degradation, crop safety concerns, and environmental quality deterioration. Advanced technologies, including electrochemical detection, optical sensing, and biosensing, enable precise and real-time monitoring of soil pollutants (Garlando et al., 2020). Electrochemical sensors quantify changes in soil conductivity due to specific contaminants, while optical sensors utilize light interactions to identify pollutants, such as organic chemicals. Biosensors employ biological components, including enzymes or microbes, for the precise detection of toxic substances. These sensors are employed in multiple applications, such as monitoring soil health in agriculture, optimizing fertilizer use, ensuring environmental compliance, and aiding soil remediation efforts. Soil pollutant sensors enable the early identification of contaminants, which mitigates risks to human health, protects ecosystems, and promotes sustainable agricultural practices. Their role is essential in addressing soil contamination problems (Garnaik and Nayak, 2024).

### 2.3 Insect/pest sensors

Plant diseases and pests can significantly compromise the quality and yield of agricultural products by inflicting damage on plant roots, bulbs, and aerial parts through their feeding behaviors. Common soil-dwelling pests that contribute to agricultural loss include various species such as beetles, moths, butterflies, and flies. To facilitate the detection of these soil pests, a range of advanced methodologies has been developed. Optoelectronic sensors harness light-based technologies to identify changes in environmental conditions attributable to pest activity. Acoustic sensors capture the sounds generated by pests while they interact with plants or soil, thereby enabling their identification. Impedance sensors assess variations in electrical resistance that may indicate the presence of pests or their feeding behavior (Stańczyk et al., 2023).

Furthermore, nanostructured biosensors provide a highly sensitive detection mechanism by utilizing nanomaterials to enhance their capabilities, facilitating the identification of specific pests or biological markers associated with pest-induced damage. These innovative detection methodologies furnish agricultural practitioners with essential tools to monitor and manage pest populations more effectively, ultimately contributing to the protection of crop health and the enhancement of agricultural productivity.

Fazeel Ahmed Khan and his team have developed an IoT-based system for environmental monitoring and disease detection in smart greenhouses. The system monitors the greenhouse’s environment, manages water irrigation, collects images, and predicts plant diseases using leaf datasets. The research validates the proposed system design and architecture for IoT-based monitoring and water irrigation management. The system also enhances greenhouse management and supports agribusinesses and farmers by transitioning traditional greenhouses into smart greenhouses, thereby automating and improving agricultural practices using advanced technologies (Khan et al., 2020).

### 2.4 Plant stress sensors

Plant stress refers to the negative impact on plant growth and development due to biotic and abiotic factors, such as pests, diseases, drought, salinity, and extreme temperatures (Bashir et al., 2021). Understanding and managing plant stress is crucial for improving crop yield and sustainability, especially in the face of global challenges like climate change and food security issues (Galieni et al., 2021; Mansoor et al., 2022). Plant stress mechanisms involve complex physiological and biochemical processes, triggering a cascade of molecular and cellular responses. Techniques for detecting plant stress include remote sensing, thermal imaging, fluorescence imaging, and spectroscopy and hyperspectral imaging. Types of stress sensors include moisture sensors, nutrient sensors, soil salinity sensors, gas exchange sensors, and chlorophyll fluorescence sensors. Moisture sensors monitor soil and plant water status, while nutrient sensors detect deficiencies or toxicities of nutrients. Soil salinity sensors measure soil salinity, which can adversely affect plant growth due to osmotic stress and nutrient imbalance (Yin et al., 2021). Gas exchange sensors measure photosynthesis and respiration rates, indicating plant health and stress levels. Future directions in plant stress detection include Positron Emission Tomography (PET) and advanced metabolomics, which offer deeper insights into physiological and metabolic changes occurring in plants under stress (Galieni et al., 2021). These technologies provide more detailed data and non-destructive ways to monitor plants, facilitating timely interventions and better management practices to enhance plant health and crop yields (Yin et al., 2021; Galieni et al., 2021).

### 2.5 Positional and motion sensors

Motion and positional sensors are critical tools in precision agriculture, enhancing the implementation of farming practices. These sensors enable precise navigation and guidance of agricultural machinery, ensuring the accurate execution of tasks such as planting, fertilizing, and harvesting. The integration of GPS and IoT technologies facilitates the creation of comprehensive field maps, the tracking of machinery movement, and the enhancement of automated systems, including tractors and drones (Pandey et al., 2021; Getahun et al., 2024). The application of variable rate technology (VRT) through these sensors allows farmers to apply precise amounts of seeds, fertilizers, or water in designated areas, thus reducing waste and improving efficiency (He, 2023). They enhance sustainability by reducing fuel consumption, limiting chemical overuse, and decreasing environmental impact. Motion and positional sensors are critical for tracking livestock movement and protecting valuable agricultural assets (Tenreiro et al., 2023). The ability to provide real-time feedback and data-driven insights allows farmers to make informed decisions, enhancing productivity, lowering costs, and fostering sustainable agricultural practices. With the advancement of precision agriculture, these sensors play a crucial role in improving accuracy and efficiency in modern farming practices (Nackley et al., 2021; Rajak et al., 2023; Naidu et al., 2024).

## 3 IoT and sensor integration in precision agriculture

The IoT is revolutionizing agriculture by enabling smarter resource management and enhancing productivity. IoT-based systems use intelligent sensors to monitor field conditions in real-time, transmitting data via wireless networks to cloud platforms for precise irrigation adjustments (Rajak et al., 2023). IoT integration with mobile internet allows farmers to remotely monitor and control agricultural systems using mobile applications. Combining IoT with agricultural robotics advances intelligent farming practices, with robots autonomously performing tasks like seeding, fertilization, and pesticide application (Botta et al., 2022).

IoT platforms also facilitate environmental monitoring and early pest detection, with tools like video surveillance and pest-monitoring lamps enabling remote observation of pest activity. Meteorological data collected by IoT sensors aids in forecasting agricultural disasters. However, challenges like high costs, inconsistent standards, and limited compatibility across platforms hinder widespread IoT adoption in agriculture. Addressing these barriers by developing unified data standards and cost-effective IoT products can enhance agricultural productivity, expand benefits to more farmers, and promote sustainable agricultural development (Xu et al., 2022).

The integration of IoT with diverse sensor technologies allows for continuous, real-time monitoring of various agricultural parameters such as soil moisture, pH levels, temperature, nutrient status, and plant health (Marios and Georgiou, 2017; Khanal et al., 2020). These sensors collect data at high temporal and spatial resolutions, providing a detailed view of the field conditions. The real-time data transmission enabled by IoT technologies ensures immediate availability of information, which is crucial for timely decision-making and intervention (Wang et al., 2011). IoT-enabled sensor networks offer the capability for remote monitoring of agricultural fields, reducing the necessity for physical presence (Srbinovska et al., 2015).

Through web interfaces and mobile applications, farmers can access data collected by sensors from anywhere, enabling them to monitor and manage their fields more effectively. This remote accessibility is particularly beneficial for large-scale operations or farms located in hard-to-reach areas (Hashim et al., 2015). Integrating sensors with IoT in agriculture offers significant benefits, including process automation driven by real-time data. For instance, irrigation systems can adjust automatically based on soil moisture levels, while fertilizer and pesticide applications can be tailored to the specific needs of different crop zones, reducing waste and environmental impact (Liang and Shah, 2023). IoT platforms aggregate data from various sensors, enabling advanced analytics and predictive insights that help farmers anticipate issues like pest outbreaks and plant diseases. Furthermore, ML algorithms optimize resource allocation and crop management by leveraging historical and real-time data (Mowla et al., 2023).

The scalable design of IoT platform allows for the integration of diverse sensors and data sources, ensuring adaptability to changing farm conditions and facilitating a flexible management approach in precision agriculture (Sharma and Shivandu, 2024). The use of IoT technology in precision agriculture is markedly reducing human involvement while improving operability and system stability. IoT-driven smart irrigation systems, autonomous machinery, and real-time soil and crop monitoring facilitate efficient resource utilization and enhanced productivity. Soil moisture sensors and automated irrigation optimize water consumption, while GPS-guided tractors, drones, and robotic seeders execute field operations autonomously (Shantaram et al., 2005; Pratama et al., 2021).

AI-driven predictive maintenance guarantees equipment dependability by identifying problems before to failure (McCole et al., 2023). Furthermore, cloud-based farm management systems provide farmers with remote monitoring and decision-making capabilities, including real-time sensor data, weather predictions, and AI analytics. The use of 5G, LoRaWAN, and edge computing enhances system connectivity and reactivity, allowing fully autonomous, data-driven agriculture. These developments result in reduced labor costs, optimal resource use, and improved sustainability, guaranteeing that the future of precision agriculture is more efficient, resilient, and ecologically sustainable (Liu et al., 2024).

## 4 Smart sensors and IoT in precision agriculture

**Figure 3:**
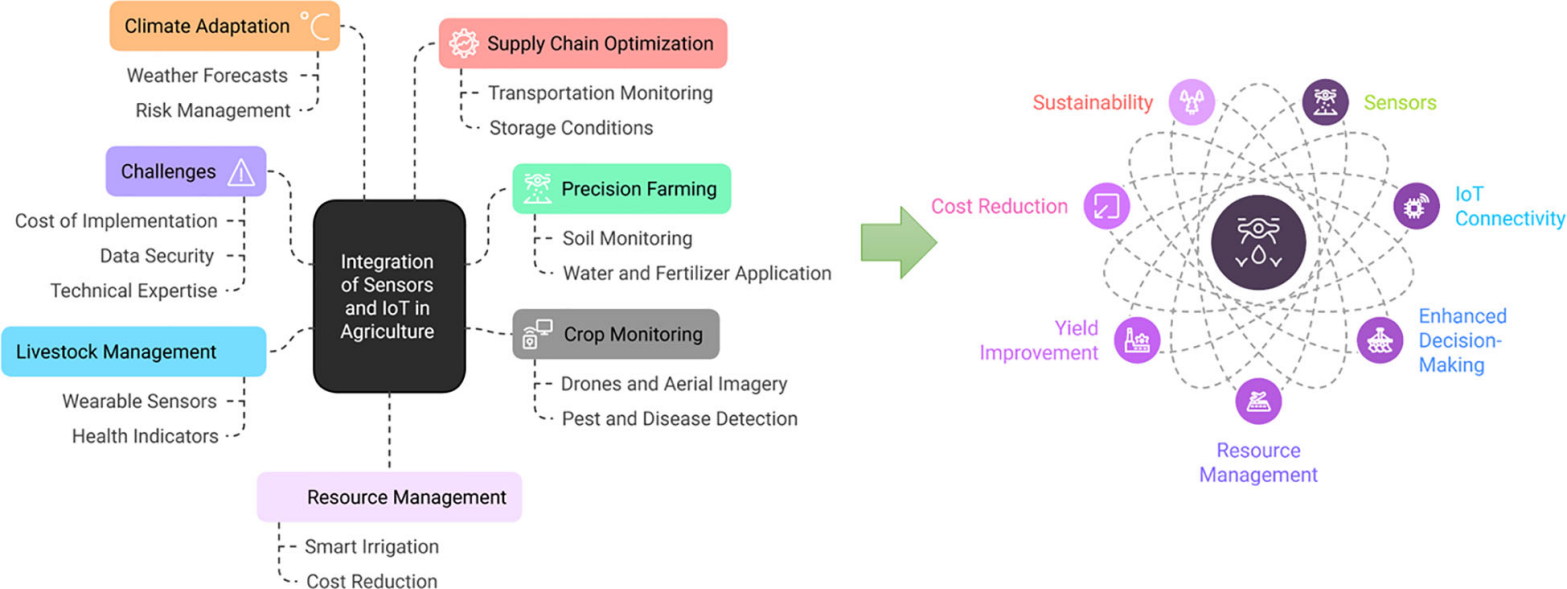
Figure highlights key applications including resource efficacy, data-driven decision-making, livestock monitoring, precision farming, pest and disease management, smart irrigation, greenhouse automation, and supply chain optimization. Sustainability, cost reduction, yield improvement and resource management can enhance productivity, sustainably manage re-sources, and improve agricultural efficiency.

Modern technology is important in maintaining agricultural productivity even with limited resources. It helps farmers monitor climate changes, track soil nutrient levels, manage water usage, and streamline data handling in farming operations. Various sensors and computing tools are now available to collect and manage data from cropping systems to make timely and informed decisions (Ali et al., 2023). Various digital platforms and camera-based monitoring systems empower farmers to observe their fields remotely. IoT applications and smart farming techniques enhance decision-making by simulating and forecasting crop yields under anticipated climatic conditions (Akbar et al., 2024). Moreover, advanced neural networks and simulation models have reliable decision support for farming activities (**Figure 3**). By integrating these technologies, farmers can optimize resource use, minimize waste, and enhance crop health and productivity, contributing to more sustainable and efficient farming systems (Mana et al., 2024).

**Figure 4:**
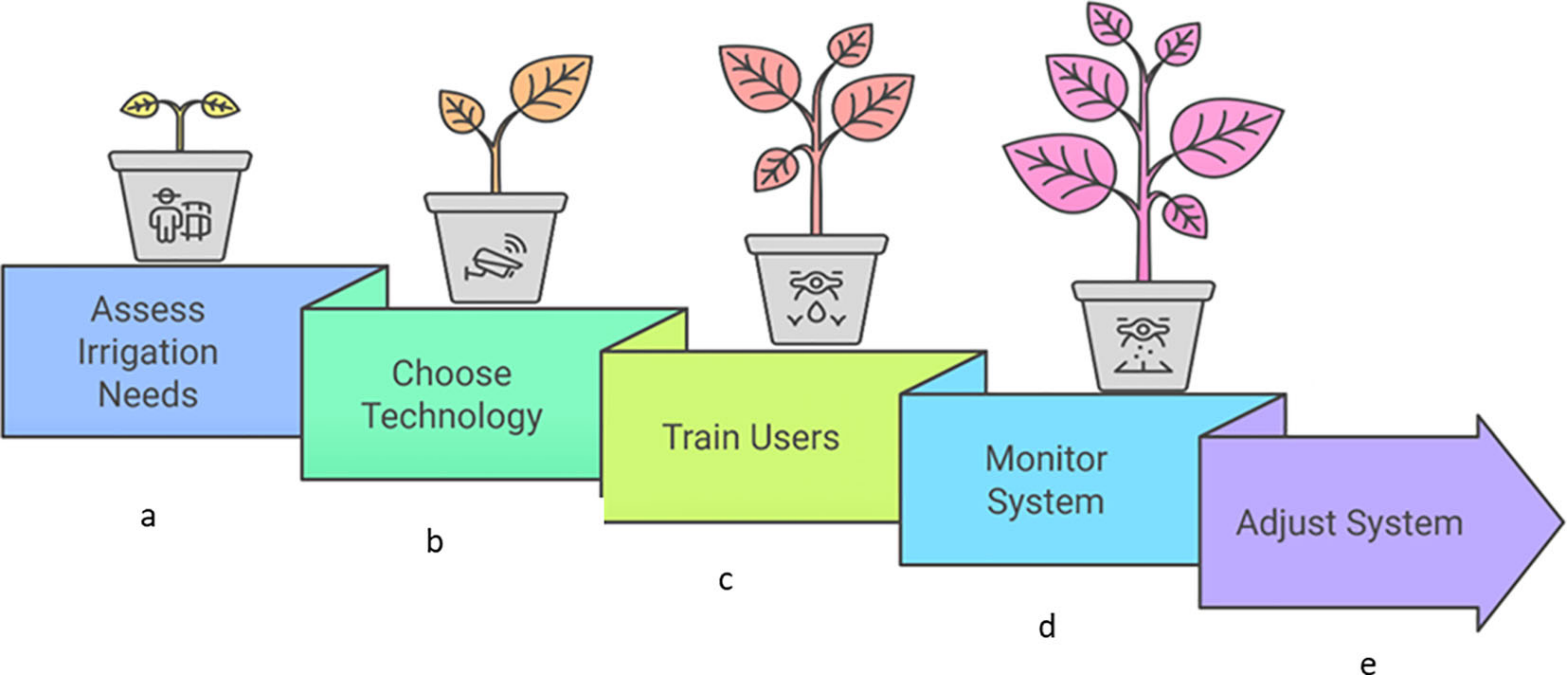
It explains a five-stage process for implementing smart irrigation systems. The figure depicts a sequential workflow for establishing effective smart irrigation. This involves: **(a)** Evaluating site-specific irrigation requirements. **(b)** Selecting appropriate sensors and controllers. **(c)** Providing training on system operation. **(d)** Continuously checking system performance. **(e)** Making necessary adjustments for efficiency. Each stage is visually represented by a potted plant progressing in growth, illustrating the impact of effective irrigation management.

Precision farming uses IoT-enabled sensors to monitor and manage agricultural operations with the highest accuracy. Various sensors embedded in fields collect data on soil moisture, pH, temperature, and nutrient levels. These sensors provide farmers with real-time data for the health and needs of various crops. The biggest advantage of this granular data includes the optimization of irrigation, fertilization, and pest control resulting in increased yields and reduced resource wastage (Sishodia et al., 2020). For example, soil moisture sensors can help farmers determine the optimal watering schedule, preventing over-irrigation and conserving water (**Figure 4**). Similarly, nutrient sensors analyse soil composition to recommend precise fertilizer applications, reducing costs and minimizing environmental runoff. Precision farming technologies also give variable-rate application of inputs which helps to treat specific areas of a field differently based on their unique conditions. This level of customization improves productivity while promoting environmental sustainability (Sishodia et al., 2020).

IoT and sensors have transformed livestock management by continuous monitoring of animal health, behavior, and the environment. Some wearable sensors on livestock collect data on body temperature, movement, heart rate, and feeding habits. This information is then transmitted to centralized platforms where farmers can identify health issues or various irregularities (Isaac, 2021; Monteiro et al., 2021). For example, sensors can detect signs of illness, such as reduced movement or abnormal temperature. Additionally, sensors monitor reproductive cycles showing timely breeding and increasing reproduction rates. IoT platforms can also track the location and activity of grazing animals, reducing the risk of theft and improving pasture management. This combination of real-time monitoring and predictive analytics helps increase animal welfare, boost productivity, and reduce economic losses (Davaji et al., 2017).

### 4.1 Smart irrigation systems

Water is one of the most critical resources in agriculture and its efficient use is important in growing scarcity (Mansoor and Chung, 2024). IoT-enabled smart irrigation systems provide a solution by automating water distribution based on real-time data. Sensors placed in fields monitor soil moisture, weather conditions, and crop water requirements. These systems then analyze the data to activate irrigation systems only when needed. Smart irrigation reduces water consumption by preventing overwatering and ensuring even distribution (**Figure 4**). For example, drip irrigation systems equipped with IoT sensors deliver water directly to plant roots. This minimizes evaporation and runoff (Math et al., 2018).

Water scarcity is a significant challenge for global agriculture, necessitating the adoption of smart irrigation systems that utilize real-time data from soil moisture sensors, weather conditions, and crop water requirements to optimize water use efficiency (Ingrao et al., 2023) Traditional irrigation methods often lead to water wastage, uneven distribution, and reduced crop yields due to inefficient scheduling and overwatering. IoT-enabled smart irrigation systems integrate wireless sensors, cloud computing, and AI to automate water distribution based on real-time environmental conditions, ensuring crops receive the optimal amount of water while minimizing losses due to evaporation and runoff (Koul et al., 2022).

Soil moisture sensors provide continuous feedback on volumetric water content (VWC) and soil matric potential. Weather monitoring systems allow dynamic adjustments to irrigation schedules, while evapotranspiration models predict water loss through transpiration and soil evaporation. The optimization of smart irrigation is achieved through various adaptive techniques that integrate sensor data with AI-based decision models (Veeramanju, 2024).

Real-time irrigation optimization has been shown to yield significant water savings while improving crop productivity. Studies indicate that smart irrigation can reduce agricultural water consumption by 30-50% compared to conventional practices (Mallareddy et al., 2023). AI-based irrigation scheduling improves water-use efficiency (WUE) by up to 60%, ensuring that each unit of water applied contributes maximally to crop growth and yield. Smart irrigation also prevents soil erosion and nutrient leaching, preserving soil fertility and long-term sustainability (Alharbi et al., 2024).

As technology continues to evolve, the future of smart irrigation systems looks promising. Innovations such as AI and ML are expected to enhance the predictive capabilities of these systems, allowing for even more precise irrigation management (**Figure 4**). Additionally, the increasing focus on sustainable agriculture and water conservation will likely drive the adoption of smart irrigation technologies worldwide (Zhang et al., 2021; Sharma and Shivandu, 2024). Smart irrigation systems are transforming the way we manage water resources in agriculture. By leveraging technology to optimize irrigation practices, these systems not only conserve water but also promote sustainable farming and enhance crop productivity. As we move towards a future where water scarcity is a growing concern, the importance of smart irrigation systems will only continue to rise. The effectiveness of these systems hinges on various sensor types, each with a specific function, and application. As water scarcity, drought, climate changes intensify, the importance of smart sensor systems will continue to grow.

### 4.2 Pest and disease management

Crop losses due to pests and diseases are a significant challenge for farmers. Various sensors installed in fields can detect environmental conditions that favour pest infestations or disease outbreaks, such as high humidity or temperature fluctuations (Wang et al., 2024). Additionally, advanced imaging sensors can identify early signs of plant stress or damage caused by pests. IoT networks connect these sensors to central platforms which analyze the data and send alerts to farmers. This early warning system leads to targeted interventions, such as applying pesticides or introducing biological control agents (Wang et al., 2024).

Crop health sensors and smart pest traps provide real-time data on plant stress and pest populations, allowing for early detection of pests and diseases. This data is analyzed using advanced algorithms and farm management software, enabling predictive analytics and threshold alerts for timely intervention. By targeting specific problem areas, farmers can implement focused control measures, thereby minimizing the use of chemical pesticides. This approach supports Integrated Pest Management (IPM) and enhances sustainable farming practices, ultimately promoting healthier crops and reducing environmental impact. IPM emphasizes the use of monitoring and assessment to identify pest species and their life cycles, enabling targeted and timely interventions. IPM utilizes a mix of biological control, cultural practices, habitat manipulation, and, when necessary, chemical methods in a way that minimizes risks to human health, beneficial organisms, and the environment. By fostering ecological balance and promoting natural pest resistance, IPM seeks to reduce reliance on chemical pesticides and improve the long-term health of ecosystems. This approach is particularly relevant in agriculture but is also applicable in urban settings and natural resource management, making it a valuable framework for sustainable pest management (Khan et al., 2020; Chin et al., 2023).

### 4.3 Supply chain optimization

The benefits of IoT in agriculture extend beyond the farm, revolutionizing the agricultural supply chain. Smart sensors track the journey of produce from the field to the market. This ensures quality control and traceability. For example, temperature and humidity sensors in storage and transport facilities monitor conditions for perishable goods that reduce spoilage and ensure freshness. IoT platforms also provide real-time updates on the location and status of shipments thereby improving delivery times. This transparency is best for consumer confidence in the safety and authenticity of agricultural products (Shiyale et al., 2020). Supply chain optimization is a critical aspect of modern business strategy, aiming to enhance efficiency and effectiveness from raw material procurement to product delivery to the end customer. The initial step in this process involves comprehensive data collection and analysis, which provides a foundation for understanding current performance. By gathering data from procurement, production, inventory, and distribution, businesses can identify bottlenecks and areas for improvement. Utilizing advanced analytics tools aids in diagnosing issues and setting the stage for continuous improvement. Following data analysis, accurate demand forecasting becomes imperative. Leveraging historical data alongside market trends allows organizations to predict market demand with greater precision, thereby reducing the likelihood of overproduction or shortages. Advanced forecasting methods enable dynamic adjustments, informed by real-time data, ensuring that businesses remain agile and responsive to market changes.

Inventory management plays a crucial role in supply chain optimization by balancing stock levels to avoid both overstock and stockouts. Techniques such as just-in-time (JIT) inventory or safety stock calculations help maintain this balance. Efficient inventory management reduces holding costs and improves cash flow while ensuring product availability. In optimizing supplier relationships, businesses need to focus on collaboration and effective communication. Building strong relationships with suppliers is essential for negotiation and reliability. Developing supplier scorecards can help evaluate performance, fostering a culture of continuous improvement and innovation. These relationships are pivotal in creating a resilient supply chain. Process and workflow improvements are another critical component. Streamlining operations by eliminating waste and redundancies can significantly enhance productivity.

Methodologies such as Lean, Six Sigma, or Total Quality Management (TQM) are valuable in identifying inefficiencies and driving process enhancements, ultimately leading to increased operational efficiency. Technology integration offers transformative potential in supply chain optimization. Implementing supply chain management software and automation tools can increase visibility and coordination across the supply chain. Emerging technologies like the Internet of Things (IoT), Artificial Intelligence (AI), and Blockchain provide opportunities for real-time tracking and secure transactions, further enhancing supply chain efficiency.

**Figure 5:**
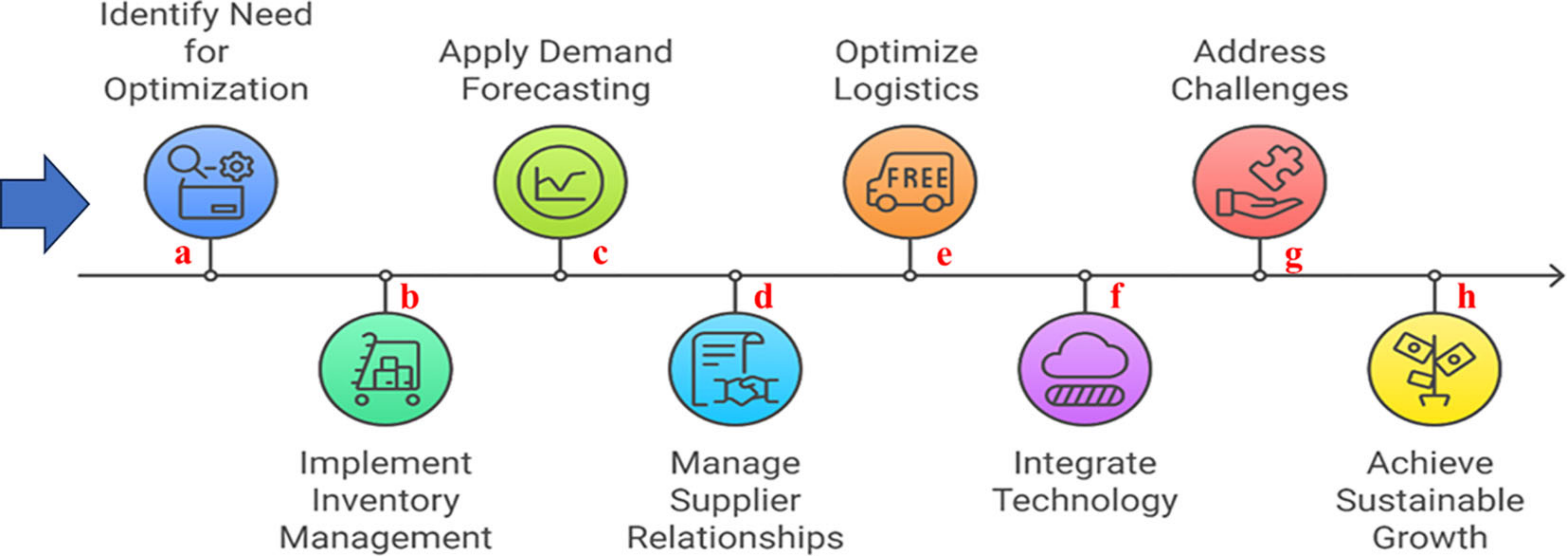
This figure illustrates key strategies for optimizing supply chains, which include **(a)**, Identification of need **(b)** Inventory Management, **(c)** Demand Forecasting, **(d)** Supplier Relationship Management, **(e)** Logistics Optimization, **(f)** Technology Integration, **(g)** addressing challenges and **(h)** achieve growth by enhance overall supply chain performance. Each strategy plays a critical role in enhancing efficiency, reducing costs, and improving customer satisfaction within the supply chain framework.

Logistics and distribution optimization are vital for minimizing costs and delivery times. By planning optimal transportation routes and utilizing distribution centers strategically, businesses can ensure rapid delivery and reduced logistics costs. This logistical agility is crucial in today’s fast-paced business environment, where customer expectations for quick delivery are high (**Figure 5**).

Risk management and contingency planning are essential for safeguarding against potential disruptions such as supplier failures or demand fluctuations. Identifying these risks and developing comprehensive contingency plans ensures that businesses can maintain operations even in adverse conditions, thereby increasing supply chain resilience. Finally, performance measurement and continuous improvement are necessary to sustain supply chain optimization. Establishing key performance indicators (KPIs) provides a benchmark for evaluating success. Regularly reviewing and refining strategies based on KPI performance ensures that the supply chain remains efficient and aligned with organizational goals. Engaging in cross-functional collaboration and enhancing communication channels within the organization further supports this ongoing optimization process. Supply chain optimization is essential for businesses aiming to enhance their operational efficiency and customer satisfaction. By implementing effective strategies and leveraging technology, organizations can navigate the complexities of supply chains and achieve sustainable growth. Continuous assessment and adaptation to market changes will further ensure that supply chains remain resilient and competitive.

### 4.4 Greenhouse automation

IoT-enabled sensors and actuators automate greenhouse operations for the optimal conditions for plant growth. Sensors monitor variables such as temperature, humidity, light intensity, and carbon dioxide levels, while actuators adjust ventilation, heating, and lighting systems accordingly. For example, if sensors detect high temperatures, IoT platforms can automatically activate cooling fans or open vents to regulate the environment (Mishra et al., 2025). This level of automation reduces labour costs, maintains consistent growing conditions, and maximizes yields. Greenhouse automation is an innovative approach in modern agriculture that significantly enhances productivity and sustainability by integrating various technologies. Greenhouse automation offers significant advantages for modern agriculture, leading to enhanced environmental control, increased labor efficiency, data-driven decision-making, optimized resource utilization, and ultimately, higher crop yields (Shiyale et al., 2020; Acharya et al., 2022). This technology leverages several key components, including climate control systems for precise management of temperature, humidity, and ventilation, automated irrigation systems for efficient water distribution, sophisticated lighting systems to optimize light exposure, and automated nutrient delivery systems for precise feeding (Kumar et al., 2022a). Additionally, a network of monitoring sensors collects real-time data on various environmental parameters and plant health.

#### 4.4.1 Climate control systems

- *Temperature Management:* These systems employ heaters, coolers, and fans to maintain optimal temperature ranges for plant growth.
- *Humidity Control:* By utilizing dehumidifiers and humidifiers, these systems help maintain appropriate moisture levels, which is vital for plant health.
- *Ventilation:* Automated vents and exhaust systems regulate airflow, preventing overheating and ensuring fresh air circulation.

#### 4.4.2 Automated irrigation systems

- *Drip or Sprinkler Irrigation:* These systems allow for precise water delivery directly to plant roots or over crop surfaces, minimizing water waste.
- *Soil Moisture Sensors:* These sensors monitor soil moisture levels, enabling irrigation to be scheduled based on real-time data rather than a fixed schedule.

#### 4.4.3 Sophisticated lighting systems

- *LEDs and Grow Lights:* These specialized lights provide optimal wavelengths for photosynthesis and can be controlled to simulate natural light cycles, promoting healthy plant growth.
- *Light Intensity and Duration Control:* Automated systems adjust light intensity and exposure duration depending on plant needs and growth stages.

#### 4.4.4 Nutrient delivery systems

- *Fertigation Systems:* These automatically mix fertilizers with irrigation water, ensuring plants receive nutrients in the right proportions at the right times.
- *Monitoring Nutrient Levels:* Sensors can measure nutrient concentrations in the substrate, allowing for adjustments in real-time.

#### 4.4.5 Monitoring sensors and data collection

- *Environmental Sensors:* These devices collect data on temperature, humidity, light intensity, CO2 levels, and more, providing a comprehensive view of the greenhouse environment.
- *Plant Health Monitoring:* Sensors can track plant growth metrics and detect stress indicators, allowing for timely interventions.

#### 4.4.6 Implementation strategy

- *Needs Assessment:* Conduct a thorough evaluation of the greenhouse’s current capabilities and limitations to determine which automation features are most beneficial.
- *Technology Selection:* Choose technologies and systems that align with the greenhouse’s specific requirements while ensuring compatibility with existing infrastructures.

Successful greenhouse automation hinges on an iterative process of evaluation and adaptation. Regular data analysis allows growers to identify areas for improvement, streamline processes, and incorporate new technologies as they become available (Kumar et al., 2022b). This ongoing commitment to enhancement not only maximizes immediate benefits but also prepares the greenhouse for future challenges and opportunities. By understanding and implementing these components and strategies, growers can fully realize the advantages of greenhouse automation, ensuring more efficient and sustainable agricultural practices (Acharya et al., 2022). Greenhouse automation presents numerous opportunities for innovation and growth in the agricultural sector. By embracing these technologies, farmers can enhance productivity, reduce costs, and contribute to a more sustainable food system. As the industry continues to evolve, those who invest in automation will likely lead the way in meeting the challenges of modern agriculture.

### 4.5 Decision-making, resource efficiency and sustainability

The vast amounts of data generated by sensors and IoT devices enable advanced analytics and AI applications in agriculture. Farmers can use this to make informed decisions about planting schedules, crop rotation, and resource allocation. Predictive analytics can forecast weather patterns, pest outbreaks, and market trends. AI-driven systems analyze historical and real-time data to recommend optimal farming practices (Qazi et al., 2022). For example, ML algorithms can identify patterns in soil and weather data to suggest the best times for planting and harvesting. Precision agriculture leverages technology and data analysis to improve decision-making, optimize resource efficiency, and enhance sustainability. By using various tools, such as satellite imagery, drones, sensors, and weather stations, farmers can collect vast amounts of data regarding crop health, soil conditions, and environmental factors. This data-driven insight allows for informed decisions that can lead to more effective management practices, reducing uncertainty and risk in agricultural operations (Xu et al., 2022; Hoque and Padhiary, 2024).

One of the key aspects of decision-making in precision agriculture is predictive analytics. Advanced algorithms and machine learning techniques can analyze historical and real-time data to forecast crop yields, pest outbreaks, and potential disease occurrences. This predictive capability enables farmers to take proactive management steps, thereby enhancing crop resilience. Additionally, precision agriculture promotes customized farming practices by employing variable rate technology (VRT), which allow farmers to tailor inputs—such as seeds, fertilizers, and pesticides—to specific areas in their fields based on need rather than applying uniform treatments across the entire area (Bwambale et al., 2022; Kanimozhi and Vadivel, 2024). Resource efficiency is another significant advantage of precision agriculture. By facilitating precise application of fertilizers, pesticides, and water, farmers can optimize input costs while maximizing effectiveness. For example, by using soil moisture sensors and automated irrigation systems, water can be applied only where and when necessary, which is crucial especially in regions facing water scarcity. Moreover, GPS-guided machinery ensures accurate planting, cultivation, and harvesting, thereby reducing fuel consumption and wear on equipment. This attention to resource management not only saves costs but also minimizes environmental impacts, particularly regarding chemical runoff and waste (Pandey et al., 2021; Xu et al., 2022; Naidu et al., 2024). Sustainability is a fundamental principle underlying precision agriculture. The approach encourages practices that enhance soil health, such as cover cropping, reduced tillage, and crop rotation, all while monitoring soil conditions over time (Ullo and Sinha, 2021; Liu et al., 2022). This focus on maintaining soil quality is crucial for long-term agricultural productivity. Moreover, by precisely targeting resource applications, farmers can significantly decrease the use of pesticides and herbicides, helping preserve local ecosystems and promote biodiversity within farming landscapes. This sustainable approach can effectively lower the carbon footprint of agricultural operations, as efficient resource use translates to reduced greenhouse gas emissions (Ehsani et al., 1999).

## 5 Integration of IoT sensors with AI and ML

The integration of IoT sensors with AI and ML is revolutionizing precision agriculture by enabling real-time data collection, predictive analytics, and automated decision-making (Zhang et al., 2021; Hoque and Padhiary, 2024). IoT sensors deployed across agricultural fields continuously monitor and transmit real-time environmental and crop data, collecting information on key parameters such as soil moisture and pH, temperature and humidity, light intensity, leaf chlorophyll content, and NDVI. These sensors communicate data wirelessly via long range wide area network (LoRaWAN), 5G, or satellite networks, feeding it into AI-powered cloud platforms for further processing (Xu et al., 2022).

AI algorithms process the collected data to identify trends, detect anomalies, and make predictions. Key applications include ML for crop yield prediction, pest and disease detection, smart irrigation and water management, VRT, and decision tree algorithms for resource allocation and precision farming (He, 2023). Real-time decision-making and automated control are possible through AI and ML models continuously analyzing IoT data, allowing automated farming systems to activate smart irrigation systems, adjust greenhouse ventilation and temperature for ideal plant growth conditions, deploy autonomous drones or robotic sprayers for targeted pesticide and nutrient application, and send alerts and recommendations to farmers through mobile applications (Bwambale et al., 2022; Kanimozhi and Vadivel, 2024).

The benefits of IoT-AI integration in precision agriculture include higher crop yields, reduced costs, sustainability, labor efficiency, and climate adaptation. Accurate yield predictions and early disease detection lead to improved productivity, while precision application of resources lowers input costs. Data-driven decisions reduce waste and promote environmentally friendly farming. Automated AI-powered systems minimize manual intervention, and predictive models help farmers adjust to changing weather patterns proactively (Veeramanju, 2024).

## 6 Challenges associated with precision agriculture

The integration of advanced technologies such as AI, sensors and the IoT in agriculture presents transformative opportunities to enhance productivity and sustainability. However, the adoption of smart farming faces significant barriers that affect its successful implementation. A major obstacle in smart farming is the unclear ownership of data generated by precision agricultural technologies. Farmers produce substantial volumes of data, yet uncertainties regarding data rights, sharing practices, and usage often lead to conflicts and reluctance in adopting new technologies. Additionally, the heterogeneous nature of agricultural data necessitates proprietary software platforms for storage and transfer, further complicating ownership disputes (Wiseman et al., 2019; Demestichas et al., 2020; Saiz-Rubio and Rovira-Más, 2020).

Increased connectivity in smart farming makes systems vulnerable to cyberattacks, including data breaches and unauthorized control of autonomous machinery. Hijacking autonomous systems such as drones, robotic weeders, or tractors can result in severe disruptions, financial losses, and crop damage. The need for robust cybersecurity frameworks to safeguard data privacy and system integrity is critical to building trust and resilience in smart farming (Barreto and Amaral, 2018; Liu et al., 2020). The implementation of smart sensors also comes with a range of challenges that can hinder their effectiveness (Liu et al., 2020).

In soil monitoring, the use of soil moisture sensors and NPK sensors offers critical insights into soil health and fertility. Yet, the high initial costs of these sensors can be a significant barrier for farmers, especially small-scale operators. Additionally, NPK sensors often face challenges related to data accuracy, which can be compromised by issues like improper calibration. This often necessitates additional time and resources for regular calibration and maintenance to ensure reliable readings (Gupta et al., 2020). When it comes to crop health monitoring, multispectral cameras and drone sensors provide powerful tools for assessing crop conditions. However, the complexities associated with data processing and interpretation can overwhelm users without adequate training or resources. Moreover, consistent data management is essential to derive actionable insights; the lack of systematic approaches can lead to missed opportunities for improving crop yields. Weather tracking relies on weather stations and atmospheric sensors, yet this area faces its own set of challenges. These systems often rely on external data sources which can introduce errors into the data if the sources are unreliable. Furthermore, establishing infrastructure for atmospheric sensors in remote areas can be logistically difficult, potentially limiting the coverage and reliability of weather monitoring in sparsely populated agricultural regions (Koduru and Koduru, 2022; Otieno, 2023).

In livestock management, GPS collars and RFID tags enhance tracking and monitoring of animals, contributing to better herd management. However, concerns around privacy and data security can pose significant hurdles for adoption (Akhigbe et al., 2021). Additionally, the reliance on battery-operated devices like RFID tags presents challenges, as the effectiveness of these systems is contingent upon maintaining adequate battery life to ensure uninterrupted functionality. Supply chain efficiency through IoT-enabled GPS devices and environmental sensors contributes to streamlined operations, yet the complexity of data integration can complicate the process (Tan and Sidhu, 2022). Farmers and supply chain managers must navigate various data formats and platforms, which can lead to inconsistencies and inefficiencies. The potential for system failures in environmental sensors is another concern, as these interruptions can adversely affect operational continuity and productivity (Kleinschmidt et al., 2019; Sarma and Barbhuiya, 2019; de Araujo Zanella et al., 2020; Van Der Linden et al., 2020; Yazdinejad et al., 2021; McCaig et al., 2023).

Lastly, in the realm of sustainability, smart irrigation sensors and pH sensors represent vital innovations for resource management. However, they require ongoing maintenance and calibration to operate effectively, which can be a significant commitment for farmers. Additionally, there can be resistance from traditional farming practices, where growers may be hesitant to adopt new technologies without clear demonstrable benefits (Klerkx et al., 2019; Koduru and Koduru, 2022; Tiwari et al., 2024). Implementing IoT platforms in agriculture demands adaptability to local conditions and often requires substantial customization, making it resource-intensive. Farmers with limited technical expertise may find these systems difficult to manage, adding to implementation challenges (Gupta et al., 2020). Farmers must navigate varying regional regulations on data protection, environmental standards, and agricultural practices. Meeting these complex legal requirements can be burdensome, especially in the absence of clear guidelines (Ali et al., 2024). The absence of uniform standards for smart farming technologies creates compatibility issues among devices and platforms, hindering seamless integration (Koduru and Koduru, 2022; Otieno, 2023).

The initial investment and operational expenses for advanced technologies can be prohibitive, particularly for small-scale farmers. Limited access to affordable options exacerbates the digital divide, leaving smaller operators at a disadvantage compared to large-scale enterprises [129]. Many farmers hesitate to adopt smart technologies due to uncertain profitability. The lack of concrete evidence demonstrating financial benefits further delays widespread adoption (Tiwari et al., 2024). Limited power availability and poor connectivity in rural regions pose additional barriers to using smart farming tools. Advances in wireless power transfer and on-site energy generation are needed to mitigate these limitations. A significant knowledge gap exists between farmers and the technologies they are expected to use. While farmers possess practical expertise, many lack the specialized training required to operate sophisticated tools driven by AI and big data (Liu et al., 2020).

Ongoing education and skill development are essential for the effective use of smart farming systems. However, limited access to training programs in rural areas, along with a growing demand for skilled labor, may displace traditional agricultural workers (McCaig et al., 2023). A significant challenge with IoT devices used in smart farming is their outdoor installation, which exposes them to harsh environmental conditions such as heavy rain, dust, wind, and extreme temperatures. These adverse conditions can lead to unforeseen mechanical failures in sophisticated devices. To address this issue, manufacturers of IoT devices should utilize materials that can endure these environmental stresses, thereby enhancing the durability and reliability of their products for consistent performance over time (Rajak et al., 2023).Environmental exposure causes gradual sensor deterioration due to factors like corrosion and dust, reducing data accuracy. The limited computational capacity of agricultural sensors also makes implementing robust security measures challenging, increasing the system’s vulnerability to attacks such as sleep deprivation, which depletes battery life and disrupts data collection (Sarma and Barbhuiya, 2019).

Many farmers resist adopting new technologies due to adherence to traditional practices and skepticism about their benefits. Demonstrating tangible, long-term advantages is key to overcoming this reluctance (Van Der Linden et al., 2020) about data privacy, security, and the reliability of AI-driven decisions discourage full engagement with smart farming systems. Building trust through transparency and reliable performance is essential for widespread adoption (Kleinschmidt et al., 2019; de Araujo Zanella et al., 2020; Yazdinejad et al., 2021). Enhancing transparency and reliable performance is crucial for building trust in smart farming systems, particularly regarding data privacy, security, and AI-driven decision-making. Transparency involves clearly communicating how data is collected, stored, and utilized, allowing farmers to understand what information is being gathered and how it benefits their operations. By openly sharing details about algorithms used to make decisions, alongside robust data privacy policies that comply with regulations, stakeholders can alleviate concerns about data misuse and unauthorized access. This clarity ensures that farmers feel more secure in adopting smart farming technologies, knowing that their data is managed responsibly (**Supplementary Table S1**). Reliable performance, on the other hand, relates to the consistent accuracy and effectiveness of AI-driven technologies in improving agricultural outcomes. When farmers observe tangible benefits, such as increased yields or resource optimization, they are more likely to trust these systems (Koduru and Koduru, 2022). Providing access to performance metrics and establishing feedback mechanisms helps demonstrate the technology’s effectiveness and fosters a sense of partnership. Furthermore, offering training and support empowers farmers to use these systems competently, while creating community platforms for sharing experiences enhances mutual trust. Together, these elements form a foundation for widespread adoption of smart farming technologies, ultimately leading to more sustainable agricultural practices (Klerkx et al., 2019). While smart farming offers immense potential to revolutionize agriculture, its success depends on addressing a diverse array of technical, regulatory, economic, educational, and social barriers. Collaborative efforts among stakeholders—including farmers, technology developers, policymakers, and researchers—are vital to creating sustainable and inclusive smart farming ecosystems.

## 7 Conclusion and future

Precision agriculture, driven by the integration of sensors and the IoT, presents a transformative opportunity to enhance agricultural productivity and sustainability. In this review we explored the diverse array of sensor technologies currently employed in precision agriculture. While offering significant benefits, such as optimized resource utilization, increased yields, and improved decision-making, the widespread adoption of these technologies faces considerable challenges. High initial investment costs, the need for specialized expertise, data security concerns, and infrastructural limitations in rural areas pose significant barriers. Furthermore, the lack of clear data ownership guidelines and compatibility issues among various sensor systems and platforms hinder seamless integration.

Future for precision agriculture are promising, particularly given the ongoing advancements in AI, machine learning, and sensor technologies. Digital twins, virtual farm replicas powered by IoT data, will enable farmers to test strategies and optimize operations in simulated environments. Sustainability will be a central focus, with IoT technologies promoting carbon sequestration, minimizing resource waste, and leveraging renewable energy-powered devices. Improved connectivity through 5G networks and low power wide area network (LPWANs) will bridge gaps in rural and remote farming areas, enabling real-time monitoring and control of agricultural systems. Customizable and scalable IoT platforms will enhance affordability and usability, expanding accessibility. The development of more affordable and user-friendly systems, coupled with targeted training and education programs for farmers, will be crucial in expanding the reach and impact of precision agriculture. Addressing the data security concerns through robust cybersecurity frameworks and establishing clear data ownership protocols will foster trust and encourage wider adoption.

Practical applications in real production include automated smart irrigation, AI-powered crop health monitoring and pest control, autonomous farming equipment, supply chain optimization through blockchain and IoT, and greenhouse automation for controlled environments. These technologies provide significant economic advantages by increasing productivity, reducing input costs, and enhancing efficiency. Farmers can maximize yields while minimizing operational expenses, resulting in higher profitability and greater resilience to market fluctuations. Precision farming techniques reduce waste, conserve natural resources, and lower the carbon footprint, making agriculture more sustainable and environmentally friendly. Collaborative efforts among technology developers, policymakers, and farmers are essential to scaling up adoption of precision agriculture. Investment in affordable, user-friendly IoT solutions, standardization of sensor technologies, and education initiatives will ensure broader accessibility to smart farming practices. Establishing robust cybersecurity frameworks and clear data ownership policies will foster trust and encourage more widespread implementation.
