# Supplementary material for: Integration of smart sensors and IOT in precision agriculture: trends, challenges and future prospectives
Source: Front Plant Sci. 2025 May 14;16:1587869. doi: 10.3389/fpls.2025.1587869 (PMC12116683; doi:10.3389/fpls.2025.1587869)
Supplement: Supplementary file 1 [file Table1.docx]

Supplementary Table S1 Comparison of different sensor types, their benefits and associated challenges

| Category | Type of Sensor | Benefits | Challenges |
| --- | --- | --- | --- |
| Soil Monitoring | Soil moisture sensors | Real-time soil moisture and nutrient data | High initial costs for equipment |
|  | NPK sensors (Nitrogen, Phosphorus, Potassium) | Improved crop yields through precise irrigation | Data accuracy can be affected by sensor calibration |
| Crop Health Monitoring | Multispectral cameras | Early detection of disease and pests | Integration of various data sources |
|  | Drone sensors | Better decision-making through data analytics | Need for consistent data management |
| Weather Tracking | Weather stations | Better planning for planting and harvesting | Reliance on external data sources can lead to errors |
|  | Atmospheric sensors | Increased resilience to climate variability | Infrastructure requirements in remote areas |
| Livestock Management | GPS collars | Enhanced tracking of animal health and behavior | Privacy and data security issues |
|  | RFID tags | Optimization of feeding strategies | Dependence on battery life of wearable devices |
| Supply Chain Efficiency | IoT-enabled GPS devices | Improved logistics and inventory management | Complexity in data integration |
|  | Environmental sensors | Reduced waste and increased transparency | Potential for system failures affecting operations |
| Sustainability | Smart irrigation sensors | Efficient resource usage (water, fertilizer) | Need for ongoing maintenance and calibration of devices |
|  | pH sensors | Lower environmental impact through targeted interventions | Potential resistance from traditional farming practices |
